# Effect of dietary n‐3 PUFA supplementation on the muscle transcriptome in older adults

**DOI:** 10.14814/phy2.12785

**Published:** 2016-06-01

**Authors:** Jun Yoshino, Gordon I. Smith, Shannon C. Kelly, Sophie Julliand, Dominic N. Reeds, Bettina Mittendorfer

**Affiliations:** ^1^ Center for Human NutritionWashington University School of MedicineSt. LouisMissouri

**Keywords:** Aging, fish oil, gene expression, hypertrophy, sarcopenia

## Abstract

Dietary fish oil‐derived n‐3 PUFA supplementation can increase muscle mass, reduce oxygen demand during physical activity, and improve physical function (muscle strength and power, and endurance) in people. The results from several studies conducted in animals suggest that the anabolic and performance‐enhancing effects of n‐3 PUFA are at least in part transcriptionally regulated. The effect of n‐3 PUFA therapy on the muscle transcriptome in people is unknown. In this study, we used muscle biopsy samples collected during a recently completed randomized controlled trial that found that n‐3 PUFA therapy increased muscle mass and function in older adults to provide a comprehensive assessment of the effect of n‐3 PUFA therapy on the skeletal muscle gene expression profile in these people. Using the microarray technique, we found that several pathways involved in regulating mitochondrial function and extracellular matrix organization were increased and pathways related to calpain‐ and ubiquitin‐mediated proteolysis and inhibition of the key anabolic regulator mTOR were decreased by n‐3 PUFA therapy. However, the effect of n‐3 PUFA therapy on the expression of individual genes involved in regulating mitochondrial function and muscle growth, assessed by quantitative RT‐PCR, was very small. These data suggest that n‐3 PUFA therapy results in small but coordinated changes in the muscle transcriptome that may help explain the n‐3 PUFA‐induced improvements in muscle mass and function.

## Introduction

Dietary supplementation with fish oil‐derived n‐3 PUFA has anabolic effects in muscle and can improve physical performance in people with functional deficits. We (Smith et al. 2015) and others (Broekhuizen et al. 2005; Rodacki et al. 2012) have shown that n‐3 PUFA therapy increases muscle mass, strength and chair‐rise ability in older adults and endurance and peak work‐load during cycling exercise in persons with chronic obstructive pulmonary disease. In addition, n‐3 PUFA supplementation was found to reduce the oxygen demand during physical activity in healthy, young adults (Peoples et al. 2008; Kawabata et al. 2014). The exact cellular mechanism(s) responsible for the anabolic and performance enhancing effect of fish oil‐derived n‐3 PUFAs in people is still unclear. Studies conducted in animals (mice, rats, and piglets) suggest that the anabolic and performance enhancing effects of fish oil‐derived n‐3 PUFAs are at least in part mediated by transcriptional changes in muscle because n‐3 PUFA treatment resulted in changes in the gene expression of growth regulatory factors in muscle that are consistent with anabolism (e.g., increased *MYOD1* and *MYOG* and decreased *FOXO, MAFBX*,* MURF1*, and C5 proteasome subunit) (Castillero et al. 2009; Liu et al. 2013) and increased the gene expression of master regulators of mitochondrial function (*PPARGC1A* and *UCP3*) (Baillie et al. 1999; Mizunoya et al. 2013; Johnson et al. 2015; Philp et al. 2015). We are, however, not aware of any studies that evaluated the effect of fish oil‐derived n‐3 PUFAs on changes in the skeletal muscle transcriptome in people.

Therefore, the purpose of this study was to test the hypothesis that fish oil‐derived n‐3 PUFA therapy increases the expression of genes involved in regulating mitochondrial function and anabolic and regenerative pathways and decreases the expression of genes involved in autophagy and atrophy in muscle. Accordingly, we evaluated: (1) the expression of multiple gene set pathways known to be involved in regulating mitochondrial function, cell growth, and structural support by using the microarray technique; and (2) the gene expression of *PPARGC1A*,* PPARA*,* PDHA1*,* CPT1B, CS*,* UQCRC1*,* UQCRC2*,* COX4I1*,* COX5B*, and *UCP3* (mitochondrial biogenesis and function), and *MYOD1, MSTN, FST TIMP1, MMP14, MEGF10, GBARAP, LC3, ATOH8*, and *FOXO3, MAFBX, MURF1* (muscle growth and regeneration) using quantitative RT‐PCR in skeletal muscle biopsies of older adults who participated in a 6‐month long double‐blind, randomized controlled trial (RCT) that evaluated the effect of n‐3 PUFA therapy on muscle volume and strength (Smith et al. 2015).

## Methods

### Subjects

Muscle gene expression was examined in a subset of 20 healthy, 60–85‐year‐old men and women who participated in a larger, double‐blind RCT evaluating the effect of n‐3 PUFA therapy on muscle mass and function (Smith et al. 2015). We selected 10 subjects from the treatment group who had the largest hypertrophic response (change in thigh muscle volume) and 10 subjects from the control group who were chosen to match the subjects in the n‐3 PUFA group on age, sex, body mass index, and overall compliance to the protocol (e.g., % pills consumed). We chose this “best responder” approach to maximize the ability for detecting potentially small n‐3 PUFA‐induced changes in muscle gene expression. Written informed consent was obtained from all subjects before their participation in the study, which was approved by the Human Research Protection Office and the Clinical Research Unit Advisory Committee at Washington University School of Medicine in St. Louis, MO and registered as trial number NCT01308957 in the clinicaltrials.gov registry.

All subjects completed a comprehensive medical evaluation, which included a history and physical examination, a 75 g oral glucose tolerance test and standard blood tests. Exclusionary criteria were: body mass index ≤18.5 or ≥35.0 kg/m^2^; unstable body weight (i.e., >2 kg change during the last 6 months); exercise training (i.e., ≥1.5 h of exercise per week); serious chronic disease (e.g., cardiopulmonary disease, diabetes, chronic kidney disease, cancer); modified Physical Performance Test score <17 out of 36 (Brown et al. 2000); treatment with medications that could affect muscle mass and/or function (e.g., HMG‐CoA reductase inhibitors, corticosteroids, or androgen‐ or estrogen‐containing compounds) within 1 year before enrolling in the study; musculoskeletal or neuromuscular impairments that could interfere with exercise testing; metal implants that could interfere with magnetic resonance imaging; cognitive impairments that could interfere with obtaining informed consent, treatment adherence or testing procedures; use of tobacco products; excessive alcohol consumption (>21 and >14 units per week for men and women, respectively); consumption of >2 servings of fatty fish per week; and use of fish oil products.

### Experimental protocol

Subjects in the n‐3 PUFA group consumed four 1‐gram LOVAZA^®^ pills per day providing a total of 1.86 g eicosapentaenoic acid [20:5 n‐3] and 1.50 g docosahexaenoic acid [22:6 n‐3] per day. Subjects in the control group consumed four identical looking pills containing corn oil per day. Both, the n‐3 PUFA and corn oil pills were kindly provided by GlaxoSmithKline plc (Research Triangle Park, NC). Subjects were instructed to consume two pills in the morning with breakfast and two in the evening with dinner. Compliance was assessed by pill count at the end of the study and by changes in red blood cell fatty acid composition (Smith et al. 2015). To help ensure reliability of the pill count, subjects were given an excess number of pills and asked to return any remaining pills at the end of the study. Study endpoints were assessed before and after 6 months of treatment. All subjects were instructed to maintain their habitual physical activity and diet during the 6‐month long n‐3 PUFA and control interventions.

Thigh muscle volume, intermuscular fat content, hand grip and upper‐ and lower body 1‐repetition maximum (1‐RM) strength (i.e., the maximal amount of weight that each participant was able to lift just once), and red blood cell n‐3 PUFA content were measured during outpatient visits to the Clinical Research Unit or the Center for Clinical Imaging Research at Washington University School of Medicine as previously described (Smith et al. 2015). Briefly, thigh muscle volume and intermuscular fat content were quantified by using magnetic resonance imaging (1.5‐T superconducting magnet [Siemens, Iselin, NJ] and Matlab software [Mathworks, Natick, MA]); hand grip strength was measured using a Jamar hydraulic hand dynamometer (Patterson Medical, Warrenville, IL); 1‐RM muscle strength was evaluated by using a multi‐station weight machine (Hoist Fitness Systems, Poway, CA) for the following exercises (all bilateral): leg press, chest press, knee extension, and knee flexion; and the fatty acid profile of red blood cell lipids, which were extracted by using the Folch method (Folch et al. 1957), was determined using gas chromatography–mass spectrometry (GC‐MS; MSD 5973 System; Hewlett‐Packard). A muscle biopsy from the quadriceps femoris was obtained under local anesthesia (lidocaine, 2%) using a Tilley Henkel forceps (Smith et al. 2011a) for a minimum of 3 days after the baseline and 6‐month strength testing visits [median: 7 days, IQR: 5–9 days)]. Subjects were asked to refrain from exercise for at least 3 days and fasted for 11 ± 1 h before the biopsy procedure. The duration of fasting and time of day the biopsy was obtained was not different before and after the intervention and between the intervention and control groups. Muscle samples were rinsed in ice‐cold saline immediately after collection, cleared of visible fat and connective tissue, frozen in liquid nitrogen and stored at −80°C until final analysis.

### Gene expression analyses

#### Microarray

Microarray analyses were performed with the GeneChip Human Gene 1.0 ST array (Affymetrix, Santa Clara, CA). Raw microarray data were normalized using a robust multiarray analysis (RMA) method. To identify the pathways that were significantly altered by n‐3 PUFA therapy, normalized data were subjected to parametric analysis of gene set enrichment (PAGE) by using the R statistical software package (available at http://www.bioconductor.org) as previously described (Kim and Volsky 2005; Yoshino et al. 2011; Fabbrini et al. 2015). Pathway gene sets used in PAGE were obtained from http://www.broad.mit.edu/gsea/msigdb/msigdb_index.html (C2: curated gene sets collection). *Z* scores and *P‐*values were calculated for each gene set. Microarray data sets from this study have been deposited in the NCBI Gene Expression Omnibus (GEO) database under accession number GSE68894.

#### RT‐PCR

RNA was isolated from frozen muscle samples by using Trizol reagent (Invitrogen, Carlsbad, CA), quantified spectrophotometrically (NanoDrop 1000, Thermo Scientific, Waltham, MA) and reverse transcribed (High‐Capacity cDNA Reverse Transcription Kit, Invitrogen). Gene expression was determined using an ABI 7500 real‐time PCR system (Invitrogen) and SYBR Green Master Mix (Invitrogen) as previously described (Smith et al. 2014; Yoshino et al. 2014). The expression of each gene was determined by normalizing the cycle threshold value of each sample to the housekeeping control gene, ribosomal protein *RPLP0*. Primer details are listed in Table 1.

**Table 1:** Sequence of primers used for RT‐PCR

Gene	Accession No.		Forward (F) and reverse (R) primer
*ATOH8*	NM_032827	F:	5′‐ GCGAACGGCTATAAAACTTTCCG ‐3′
		R:	5′‐ GCACAGCAAGATGCGAGGA ‐3′
*COX4I1*	NM_001861	F:	5′‐ CAGGGTATTTAGCCTAGTTGGC ‐3′
		R:	5′‐ GCCGATCCATATAAGCTGGGA ‐3′
*COX5B*	NM_001862	F:	5′‐ ATGGCTTCAAGGTTACTTCGC ‐3′
		R:	5′‐ CCCTTTGGGGCCAGTACATT‐3′
*CPT1B*	NM_001145134	F:	5′‐ GCGCCCCTTGTTGGATGAT ‐3′
		R:	5′‐ CCACCATGACTTGAGCACCAG ‐3′
*CS*	NM_004077	F:	5′‐ TGCTTCCTCCACGAATTTGAAA ‐3′
		R:	5′‐ CCACCATACATCATGTCCACAG ‐3′
*FOXO3*	NM_001455	F:	5′‐ CGGACAAACGGCTCACTCT ‐3′
		R:	5′‐ GGACCCGCATGAATCGACTAT ‐3′
*FST*	NM_013409	F:	5′‐ ACGTGTGAGAACGTGGACTG ‐3′
		R:	5′‐ CACATTCATTGCGGTAGGTTTTC ‐3′
*GABARAP*	NM_007278	F:	5′‐ AGAAGAGCATCCGTTCGAGAA ‐3′
		R:	5′‐ CCAGGTCTCCTATCCGAGCTT ‐3′
*LC3*	NM_032514	F:	5′‐ AACATGAGCGAGTTGGTCAAG ‐3′
		R:	5′‐ GCTCGTAGATGTCCGCGAT ‐3′
*MAFBX*	NM_148177	F:	5′‐ GCCTTTGTGCCTACAACTGAA ‐3′
		R:	5′‐ CTGCCCTTTGTCTGACAGAAT ‐3′
*MEGF10*	NM_032446	F:	5′‐ GAAGACCCTAATGTGTGTAGCC ‐3′
		R:	5′‐ CAGTGCAGCTCGTGTAGTAAA ‐3′
*MMP14*	NM_004995	F:	5′‐ GGCTACAGCAATATGGCTACC ‐3′
		R:	5′‐ GATGGCCGCTGAGAGTGAC ‐3′
*MSTN*	NM_005259	F:	5′‐ TCCTCAGTAAACTTCGTCTGGA ‐3′
		R:	5′‐ CTGCTGTCATCCCTCTGGA ‐3′
*MURF1*	NM_032588	F:	5′‐ CTTCCAGGCTGCAAATCCCTA ‐3′
		R:	5′‐ ACACTCCGTGACGATCCATGA ‐3′
*MYOD1*	NM_002478	F:	5′‐ CGCCATCCGCTATATCGAGG ‐3′
		R:	5′‐ CTGTAGTCCATCATGCCGTCG ‐3′
*PDHA1*	NM_000284	F:	5′‐ TGGTAGCATCCCGTAATTTTGC ‐3′
		R:	5′‐ ATTCGGCGTACAGTCTGCATC ‐3′
*PPARA*	NM_001001928	F:	5′‐ ATGGTGGACACGGAAAGCC ‐3′
		R:	5′‐ CGATGGATTGCGAAATCTCTTGG ‐3′
*PPARGC1A*	NM_013261	F:	5′‐ TCTGAGTCTGTATGGAGTGACAT ‐3′
		R:	5′‐ CCAAGTCGTTCACATCTAGTTCA ‐3′
*TIMP1*	NM_003254	F:	5′‐ CTTCTGCAATTCCGACCTCGT ‐3′
		R:	5′‐ ACGCTGGTATAAGGTGGTCTG ‐3′
*UCP3*	NM_003356	F:	5′‐ TGTTTTGCTGACCTCGTTACC ‐3′
		R:	5′‐ GACGGAGTCATAGAGGCCGAT ‐3′
*UQCRC1*	NM_003365	F:	5′‐ GGGGCACAAGTGCTATTGC ‐3′
		R:	5′‐ GTTGTCCAGCAGGCTAACC ‐3′
*UQCRC2*	NM_003366	F:	5′‐ TTCAGCAATTTAGGAACCACCC ‐3′
		R:	5′‐ GGTCACACTTAATTTGCCACCAA ‐3′
*RPLP0*	NM_001002	F:	5′‐ GTGATGTGCAGCTGATCAAGACT ‐3′
		R:	5′‐ GATGACCAGCCCAAAGGAGA ‐3′

### Statistical analysis

Statistical analyses were carried out with SPSS (IBM, Armonk, NY). All variables were tested for normality using the Kolmogorov–Smirnov test and skewed data sets were log‐transformed for further analysis. Student's t‐test was used to compare subject characteristics between the n‐3 PUFA and control groups at baseline. Repeated measures analysis of variance (ANOVA) was used to evaluate the effect of n‐3 PUFA therapy on thigh muscle volume, intermuscular fat content, handgrip and 1‐RM muscle strength, red blood cell n‐3 PUFA content, and muscle gene expression determined by quantitative RT‐PCR. Tukey's post hoc procedure was used to locate the differences when ANOVA identified a significant group × treatment interaction. A *P*‐value of ≤0.05 was considered statistically significant. Data are presented as mean ± SEM or median [quartiles] for normally distributed and skewed data sets, respectively.

## Results

### Subject characteristics and compliance with n‐3 PUFA therapy

Baseline characteristics (age, body weight, body composition, muscle volume, intermuscular fat content, and muscle function) of subjects in the n‐3 PUFA and control groups were not different (Table 2). Average compliance with the dietary intervention, as judged by the leftover pill count, was 95.4 ± 1.3% in the n‐3 PUFA and 93.5 ± 2.0% in the control group. The red blood cell n‐3 PUFA content increased by ~140% in the n‐3 PUFA group (from 6.0 ± 0.3% to 14.0 ± 0.4% of total fatty acid content) and did not change in the control group (5.5 ± 0.4% of total fatty acid content before and 5.4 ± 0.3% after, respectively).

**Table 2:** Subject characteristics at baseline

	Control (*n* = 10)	n‐3 PUFA (*n* = 10)	*P*‐value^a^
Age (years)	70 ± 2	68 ± 2	0.60
Blood pressure			
Systolic (mm Hg)	127 ± 4	125 ± 3	0.67
Diastolic (mm Hg)	74 ± 2	73 ± 2	0.73
Plasma concentrations^b^			
Triglycerides (mmol/L)	1.01 ± 0.11	1.28 ± 0.21	0.29
HDL‐cholesterol (mmol/L)	1.48 ± 0.11	1.79 ± 0.16	0.14
LDL‐cholesterol (mmol/L)	2.92 ± 0.26	3.34 ± 0.23	0.25
Glucose (mmol/L)	5.31 ± 0.11	5.12 ± 0.14	0.30
Glucose ‐ 2 h post OGTT (mmol/L)	7.19 ± 0.47	6.41 ± 0.60	0.32
Body mass and composition			
Body mass index (kg/m^2^)	25.3 ± 1.2	26.5 ± 1.4	0.53
Body mass (kg)	74.2 ± 4.6	72.5 ± 4.5	0.80
Body fat (kg)	22.9 ± 2.8	25.7 ± 2.3	0.46
Body fat (%)	30.7 ± 3.0	35.2 ± 1.8	0.23
Thigh muscle volume (cm^3^)	3225 ± 190	3042 ± 217	0.53
Thigh inter‐muscle fat content (cm^3^)	39.3 ± 7.0	46.3 ± 3.9	0.39
Physical function			
Handgrip strength (kg)	36 ± 4	33 ± 3	0.62
Leg press, 1‐RM strength (kg)	48 ± 4	47 ± 4	0.88
Chest press, 1‐RM strength (kg)	42 ± 6	34 ± 4	0.29
Knee extension, 1‐RM strength (kg)	57 ± 9	45 ± 6	0.24
Knee flexion, 1‐RM strength (kg)	53 ± 6	44 ± 5	0.21
Sum 1‐RM strength (kg)	201 ± 23	170 ± 18	0.29

Values are mean ± SEM. OGTT, oral glucose tolerance test; 1‐RM, 1‐repetition maximum.

a Comparison between groups was performed by using Student's *t*‐test for independent samples.

b Values (except for 2 h post OGTT) were obtained after an overnight fast.

Compared with the control group, 6 months of n‐3 PUFA therapy increased thigh muscle volume by 10.3 ± 2.2% (*P* < 0.001), handgrip strength by 10.3 ± 3.3% (*P* < 0.01) and 1‐RM muscle strength by 9.1 ± 4.1% (*P* < 0.05), and lowered intermuscular fat content by 11.5 ± 5.2% (*P* < 0.05).

### Effect of n‐3 PUFA therapy on skeletal muscle gene transcription

#### Gene expression profiling by microarray analyses

Several pathways involved in regulating mitochondrial function, cell growth, and structural support were significantly affected by n‐3 PUFA therapy (Table 3). Pathways associated with respiratory electron transport and oxidative phosphorylation (mitochondrial function) and extracellular matrix (ECM) organization (structural support) were significantly increased and pathways related to calpain‐ and ubiquitin‐mediated proteolysis, mRNA translation, and inhibition of mTOR signaling were significantly decreased by n‐3 PUFA therapy (all *P* < 0.05). n‐3 PUFA therapy also increased several pathways related to G‐protein coupled receptor (GPCR) signaling (i.e., REACTOME_GPCR_LIGAND BINDING, REACTOME_GPCR_ DOWNSTREAM_SIGNALING, and REACTOME_SIGNALING_BY_GPCR; all *Z*‐scores >3.8 and *P* < 1.50E‐04), which is one of the primary pathways of n‐3 PUFA action. Additional gene set pathways in skeletal muscle that were significantly changed by n‐3 PUFA therapy can be found in the online supporting information.

**Table 3:** Gene set pathways related to mitochondrial function, growth regulation, metabolism, and structural support in skeletal muscle that were significantly changed by n‐3 PUFA therapy

Gene set name	*Z* score	*P*‐value
Mitochondrial function		
REACTOME_RESPIRATORY_ELECTRON_TRANSPORT_ATP_SYNTHESIS_BY_CHEMIOSMOTIC_COUPLING_AND_HEAT_PRODUCTION_BY_UNCOUPLING_PROTEINS_	3.60	3.16E‐04
REACTOME_RESPIRATORY_ELECTRON_TRANSPORT	3.37	7.65E‐04
KEGG_OXIDATIVE_PHOSPHORYLATION	2.96	3.10E‐03
REACTOME_TCA_CYCLE_AND_RESPIRATORY_ELECTRON_TRANSPORT	2.24	2.48E‐02
Growth regulation		
KEGG_UBIQUITIN_MEDIATED_PROTEOLYSIS	−2.13	3.29E‐02
BIOCARTA_MCALPAIN_PATHWAY	−2.59	9.66E‐03
REACTOME_ENERGY_DEPENDENT_REGULATION_OF_MTOR_BY_LKB1_AMPK	−2.80	5.15E‐03
REACTOME_TRANSLATION	−2.93	3.38E‐03
REACTOME_METABOLISM_OF_PROTEINS	−3.48	5.06E‐04
Structural support		
NABA_MATRISOME	4.26	2.05E‐05
NABA_SECRETED_FACTORS	4.19	2.77E‐05
NABA_MATRISOME_ASSOCIATED	4.05	5.01E‐05
REACTOME_EXTRACELLULAR_MATRIX_ORGANIZATION	3.36	7.65E‐04
NABA_COLLAGENS	3.25	1.14E‐03
REACTOME_PLATELET_ADHESION_TO_EXPOSED_COLLAGEN	3.22	1.28E‐03
REACTOME_NCAM1_INTERACTIONS	2.89	3.85E‐03
REACTOME_COLLAGEN_FORMATION	2.74	6.23E‐03
BIOCARTA_LYM_PATHWAY	2.59	9.46E‐03
REACTOME_INTEGRIN_CELL_SURFACE_INTERACTIONS	2.51	1.19E‐02
KEGG_ECM_RECEPTOR_INTERACTION	2.51	1.22E‐02
REACTOME_CELL_SURFACE_INTERACTIONS_AT_THE_VASCULAR_WALL	2.50	1.23E‐02
REACTOME_NCAM_SIGNALING_FOR_NEURITE_OUT_GROWTH	2.18	2.91E‐02
REACTOME_DEGRADATION_OF_THE_EXTRACELLULAR_MATRIX	1.96	5.00E‐02

Positive *Z*‐scores indicate an upregulation (increased gene expression) and negative values a downregulation (decreased gene expression) of pathways.

#### Quantitative RT‐PCR analysis of genes involved in muscle mitochondrial function, hypertrophy, atrophy, regeneration, and autophagy

**Figure 1:**
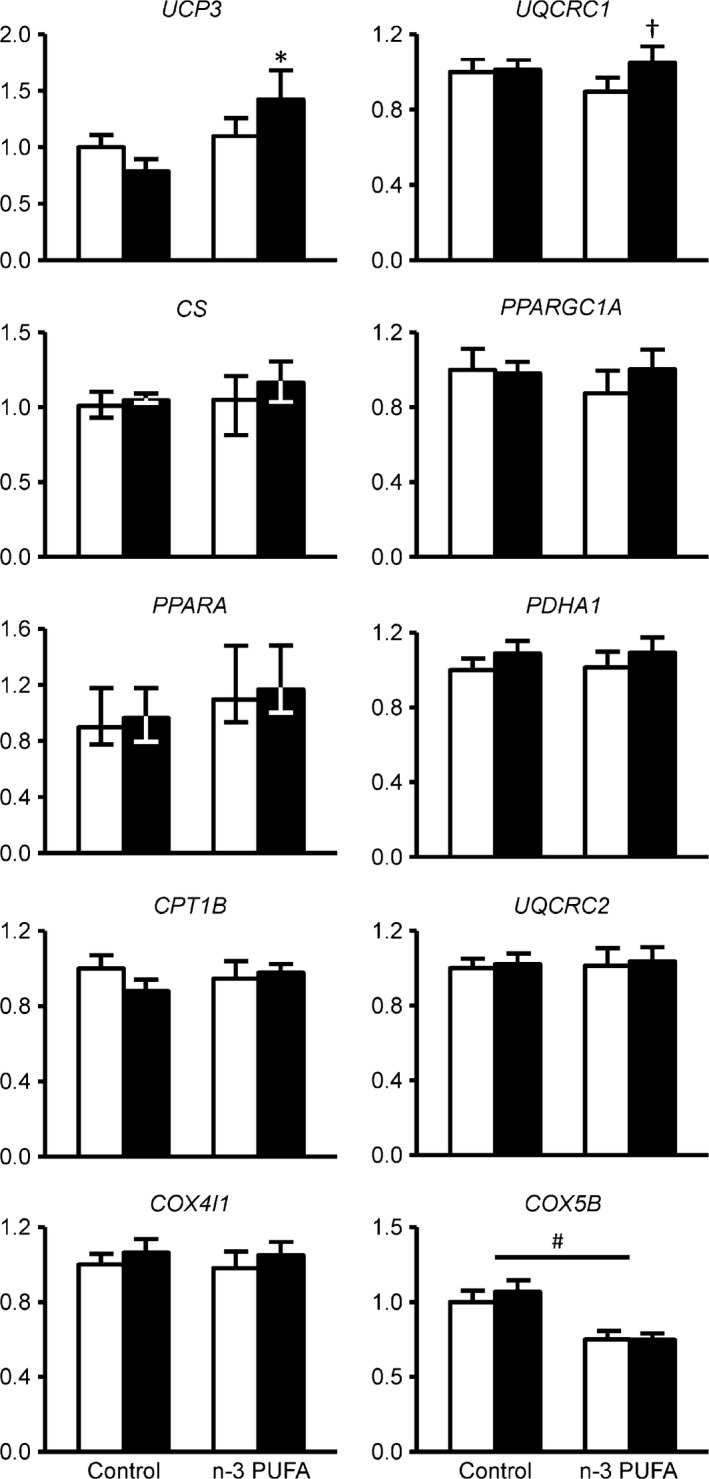
Skeletal muscle gene expression of key regulators of mitochondrial biogenesis and function. Expression of individual genes involved in mitochondrial biogenesis and function was determined by quantitative RT‐PCR before (white bars) and after (black bars) corn oil or n‐3 PUFA treatment (*n* = 10 per group). The expression of genes of interest was normalized to the expression of *RPLP0*. *Value significantly different from corresponding value in the control group (*P* < 0.05 by post‐hoc Tukey's test). ^†^Value significantly different from corresponding value before treatment (*P* < 0.01 by post hoc Tukey's test). ^#^ ANOVA revealed a significant main effect of group (*P* < 0.05). *UPC3*,*UQCRC1*,*CS*,*PPARGC1A*,*PPARA, PDHA1*,*CPT1B*,*UQCRC2*,*COX4I1,* and *COX5B* are presented as mean ± SEM; are presented as median ± quartiles. *UCP3*, uncoupling protein 3; *UQCRC1*, ubiquinol‐cytochrome c reductase core protein I; *CS*, citrate synthase*; PPARGC1A*, peroxisome proliferator‐activated receptor gamma coactivator 1 alpha; *PPARA*, peroxisome proliferator‐activated receptor alpha; *PDHA1*, pyruvate dehydrogenase (lipoamide) alpha 1; *CPT1B*, carnitine palmitoyltransferase 1B*; UQCRC2*, ubiquinol‐cytochrome c reductase core protein II;*COX4I1*, cytochrome c oxidase subunit 4I1; *COX5B*, cytochrome c oxidase subunit 5B.

**Figure 2:**
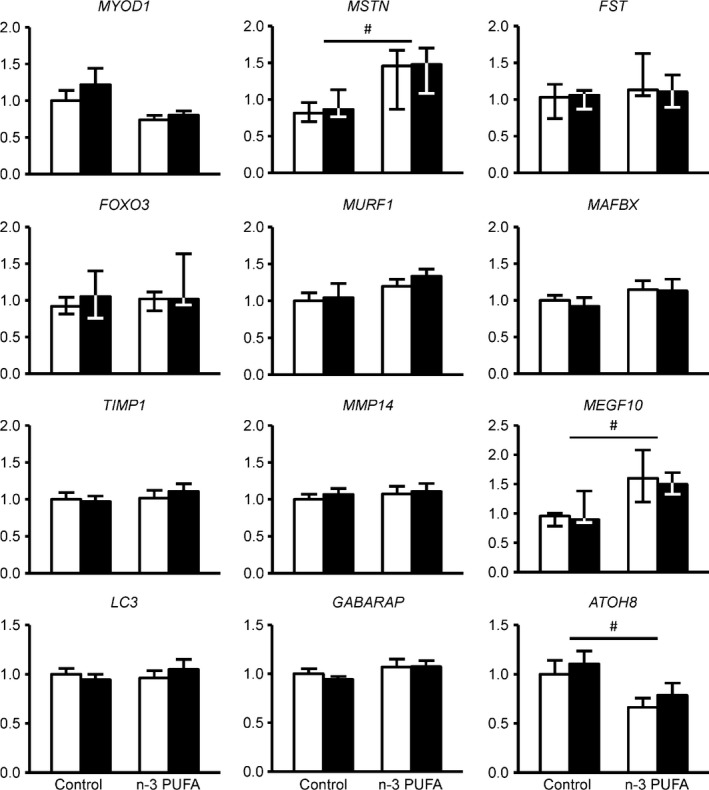
Skeletal muscle gene expression of key regulators of hypertrophy, atrophy, regeneration, and autophagy. Expression of individual genes involved muscle hypertrophy, regeneration, atrophy, and autophagy was determined by quantitative RT‐PCR before (white bars) and after (black bars) corn oil or n‐3 PUFA treatment (*n* = 10 per group). The expression of genes of interest was normalized to the expression of *RPLP0*. *MYOD1*,*MURF1*,*MAFBX*,*TIMP1*,*MMP14*,*LC3*, and *GABARAP* are presented as mean ± SEM;*MSTN*,*FST*,*FOXO3*, and *MEGF10* are presented as median ± quartiles. ^#^ ANOVA revealed a significant main effect of group (*P* < 0.05). *MYOD1*, myogenic differentiation 1; *MSTN*, myostatin; *FST*, follistatin; *FOXO3*, forkhead box O3; *MURF1*, muscle‐specific RING finger‐1; *MAFBX*, muscle atrophy F‐box; *TIMP1*, TIMP metallopeptidase inhibitor 1; *MMP14*, matrix metallopeptidase 14; *MEGF10*, multiple EGF‐like‐domains 10; *LC3*, microtubule‐associated protein 1 light chain 3 alpha; *GABARAP*, GABA(A) receptor‐associated protein; *ATOH8*, atonal homolog 8 (Drosophila).

n‐3 PUFA treatment increased (*P* < 0.05) the gene expression of *UCP3* and *UQCRC1* by ~30% and ~20%, respectively (Fig. 1). *PPARGC1A*,* PPARA*,* PDHA1*,* CPT1B, CS, UQCRC2*,* COX4I1*, and *COX5B* gene expression was unaffected by n‐3 PUFA or control oil therapy (Fig. 1). The expression of genes related to hypertrophy, atrophy, regeneration, and autophagy was not affected by n‐3 PUFA therapy and remained stable in both the n‐3 PUFA and control groups during the 6 months treatment period (Fig. 2).

## Discussion

In this study, we provide a comprehensive assessment of the effect of n‐3 PUFA therapy on the skeletal muscle gene expression profile to determine whether the beneficial effects of n‐3 PUFA on muscle mass and physical function and performance (Broekhuizen et al. 2005; Peoples et al. 2008; Rodacki et al. 2012; Kawabata et al. 2014; Smith et al. 2015) might be transcriptionally regulated. We found that several gene set pathways (assessed by microarray analyses) involved in regulating mitochondrial function and ECM organization were significantly increased and pathways related to calpain‐ and ubiquitin‐mediated proteolysis and inhibition of the key anabolic regulator mTOR were significantly decreased by n‐3 PUFA therapy; however, the effect of n‐3 PUFA therapy on the expression of a select group of individual genes involved in regulating mitochondrial function and muscle growth, assessed by quantitative RT‐PCR, was very small. These findings suggest that n‐3 PUFA therapy induces subtle but coordinated changes in key gene set pathways related to muscle structure, growth, and oxidative metabolism that may help explain the anabolic and function enhancing effects of n‐3 PUFA therapy.

Although microarray analysis indicated that several gene set pathways involved in regulating mitochondrial function were increased by n‐3 PUFA therapy, we only found a significant effect of n‐3 PUFA therapy for two genes related to mitochondrial function using the RT‐PCR technique: *UQCRC1* and *UCP3*. Several other mitochondrial genes were not affected by n‐3 PUFA treatment. The limited effect of n‐3 PUFA therapy on the expression of individual genes involved in mitochondrial function in our study is consistent with the findings from several previous studies conducted in excised rodent muscles and cultured muscle cell lines that found either a small increase (Vaughan et al. 2012; Mizunoya et al. 2013; Philp et al. 2015) or no effect (Lanza et al. 2013; Johnson et al. 2015) of n‐3 PUFA treatment on mitochondrial gene expression in cell lines and rodent models. It is possible that the effect of n‐3 PUFA may be specifically related to the electron transport chain. Both *UQCRC1* (a component of mitochondrial complex III) and *UCP3* are key parts of the electron transport chain and all of the mitochondrial pathways that were identified as being upregulated by n‐3 PUFA therapy in our microarray data set were electron transport chain related. Moreover, *fat‐1* transgenic mice, which can convert n‐6 PUFAs to n‐3 PUFAs and therefore have increased endogenous n‐3 PUFA availability, have an increased activity of complex III and IV proteins in the electron transport chain (Hagopian et al. 2010). Reduced oxidative capacity and mitochondrial dysfunction are thought to play a key role in the age‐related decline of physical function (Hiona and Leeuwenburgh 2008; Ibebunjo et al. 2013; Hepple 2014; Barbieri et al. 2015) and even a small but consistent increase in mitochondrial function genes may help explain the n‐3 PUFA‐mediated increase in endurance and oxygen demand and time to fatigue during physical activity in people (Broekhuizen et al. 2005; Peoples et al. 2008; Peoples and McLennan 2010, 2014; Kawabata et al. 2014). In addition, posttranscriptional and/or posttranslational modifications of mitochondrial gene products that affect mitochondrial protein quality are likely also involved in the beneficial effect of n‐3 PUFA therapy on physical function (Herbst et al. 2014; Johnson et al. 2015).

An increase in pathways related to ECM organization potentially provides a new mechanism for the hypertrophic and strength enhancing effect of n‐3 PUFA treatment (Smith et al. 2015) because skeletal muscle ECM provides structural integrity, regulates mechanotransduction, and coordinates cellular activities involved in muscle development, maintenance, and regeneration (Lukashev and Werb 1998; Osses and Brandan 2002; ten Broek et al. 2010; Hinds et al. 2011; Thomas et al. 2015). Indeed, changes in muscle loading that lead to muscle hypertrophy or atrophy (e.g., resistance exercise training and immobilization, respectively) are typically associated with corresponding changes in ECM‐related gene and protein expression (Cros et al. 2001; Lecker et al. 2004; Urso et al. 2006; Ogasawara et al. 2014; Hyldahl et al. 2015). Moreover, the results from studies conducted in transgenic and knockout mouse models have shown that ECM components are essential for muscle hypertrophy (Dahiya et al. 2011; Zhang et al. 2015) and anabolic signaling transduction during hypertrophy (Dahiya et al. 2011). In line with this, negative regulators of mTOR signaling were downregulated in this study and mTOR‐mediated anabolic signaling and protein synthesis were increased by n‐3 PUFA therapy in our previous study (Smith et al. 2011a). In addition, we found that calpain‐ and ubiquitin‐mediated proteolytic pathways were inhibited. Suppression of calpain and ubiquitin/proteasome pathways by n‐3 PUFA is consistent with the results obtained from a series of studies conducted in various animal models and in cultured muscle cells that demonstrate decreased mRNA expression of *MAFBX*,* MURF1*, and C5 proteasome subunit and reduced calpain and proteasome activity and rates of proteolysis after treatment with fish oil‐derived n‐3 PUFAs (Whitehouse and Tisdale 2001; Whitehouse et al. 2001, 2003; Castillero et al. 2009; Supinski et al. 2010; Huang et al. 2011; Liu et al. 2013). Accordingly, n‐3 PUFA probably exert their anabolic effect both via increasing protein synthesis and decreasing protein breakdown. The reduction of pathways related to mRNA translation in this study is most likely a reflection of increased translation efficiency, which we have demonstrated previously (Smith et al. 2011b), rather than decreased capacity for protein translation.

With the exception of *UCP3* and *UQCRC1*, we did not detect significant changes in the expression of a targeted set of individual genes (assessed by RT‐PCR) known to be involved in mitochondrial biogenesis and muscle mass regulation. It is possible that our study was underpowered to detect changes in the expression of the other genes we measured. However, we carefully selected genes that have been affected by n‐3 PUFA treatment in cell culture and animal models (Whitehouse and Tisdale 2001; Whitehouse et al. 2001; Smith et al. 2004, 2005; Castillero et al. 2009; Huang et al. 2011; Vaughan et al. 2012; Liu et al. 2013) and evaluated them in subjects who had the largest hypertrophic response (i.e., increase in thigh muscle volume) during n‐3 PUFA treatment. Therefore, it is unlikely that we did not detect biologically meaningful changes due to the small sample size or a small physiological effect size. It seems more likely that n‐3 PUFA therapy has minimal impact on the expression of individual genes in muscle, but significantly changes biological pathways that consist of a coordinated set of regulators, thereby contributing to the improvement in skeletal muscle mass and function.

In conclusion, the results from our study indicate that the beneficial effects of n‐3 PUFA on muscle mass and function/physical performance may be in part transcriptionally regulated and involves gene set pathways related to mitochondrial function, structural support, and anabolic signaling and proteolysis. Although the effect of n‐3 PUFA therapy on the expression of individual genes was small, a coordinated up‐ or downregulation of genes in specific pathways, identified by using the microarray technique, could have significant effects on muscle mass and function.

## Conflict of Interest

The authors declare no competing financial interests.

## Supporting information




**Table S1.** Additional gene set pathways in skeletal muscle that were significantly changed by n‐3 PUFA therapy.

## References

[phy212785-bib-0001] Baillie, R. A. , R. Takada , M. Nakamura , and S. D. Clarke . 1999 Coordinate induction of peroxisomal acyl‐CoA oxidase and UCP‐3 by dietary fish oil: a mechanism for decreased body fat deposition. Prostaglandins Leukot. Essent. Fatty Acids 60:351–356. 10471120 10.1016/s0952-3278(99)80011-8

[phy212785-bib-0002] Barbieri, E. , D. Agostini , E. Polidori , L. Potenza , M. Guescini , F. Lucertini , et al. 2015 The pleiotropic effect of physical exercise on mitochondrial dynamics in aging skeletal muscle. Oxid. Med. Cell Longev. 2015:917085. 25945152 10.1155/2015/917085 PMC4402202

[phy212785-bib-0003] ten Broek, R. W. , S. Grefte , and J. W. von den Hoff . 2010 Regulatory factors and cell populations involved in skeletal muscle regeneration. J. Cell. Physiol. 224:7–16. 20232319 10.1002/jcp.22127

[phy212785-bib-0004] Broekhuizen, R. , E. F. Wouters , E. C. Creutzberg , C. A. Weling‐Scheepers , and A. M. Schols . 2005 Polyunsaturated fatty acids improve exercise capacity in chronic obstructive pulmonary disease. Thorax 60:376–382. 15860712 10.1136/thx.2004.030858 PMC1758900

[phy212785-bib-0005] Brown, M. , D. R. Sinacore , E. F. Binder , and W. M. Kohrt . 2000 Physical and performance measures for the identification of mild to moderate frailty. J. Gerontol. A Biol. Sci. Med. Sci. 55:M350–M355. 10843356 10.1093/gerona/55.6.m350

[phy212785-bib-0006] Castillero, E. , A. I. Martin , M. Lopez‐Menduina , M. A. Villanua , and A. Lopez‐Calderon . 2009 Eicosapentaenoic acid attenuates arthritis‐induced muscle wasting acting on atrogin‐1 and on myogenic regulatory factors. Am. J. Physiol. Regul. Integr. Comp. Physiol. 297:R1322–R1331. 19741054 10.1152/ajpregu.00388.2009

[phy212785-bib-0007] Cros, N. , A. V. Tkatchenko , D. F. Pisani , L. Leclerc , J. J. Leger , J. F. Marini , et al. 2001 Analysis of altered gene expression in rat soleus muscle atrophied by disuse. J. Cell. Biochem. 83:508–519. 11596118 10.1002/jcb.1248

[phy212785-bib-0008] Dahiya, S. , S. Bhatnagar , S. M. Hindi , C. Jiang , P. K. Paul , S. Kuang , et al. 2011 Elevated levels of active matrix metalloproteinase‐9 cause hypertrophy in skeletal muscle of normal and dystrophin‐deficient mdx mice. Hum. Mol. Genet. 20:4345–4359. 21846793 10.1093/hmg/ddr362 PMC3196885

[phy212785-bib-0009] Fabbrini, E. , J. Yoshino , M. Yoshino , F. Magkos , C. Tiemann Luecking , D. Samovski , et al. 2015 Metabolically normal obese people are protected from adverse effects following weight gain. J. Clin. Invest. 125:787–795. 25555214 10.1172/JCI78425 PMC4319438

[phy212785-bib-0010] Folch, J. , M. Lees , and G. H. Sloane Stanley . 1957 A simple method for the isolation and purification of total lipides from animal tissues. J. Biol. Chem. 226:497–509. 13428781

[phy212785-bib-0011] Hagopian, K. , K. L. Weber , D. T. Hwee , A. L. van Eenennaam , G. Lopez‐Lluch , J. M. Villalba , et al. 2010 Complex I‐associated hydrogen peroxide production is decreased and electron transport chain enzyme activities are altered in n‐3 enriched fat‐1 mice. PLoS ONE 5:e12696. 20856881 10.1371/journal.pone.0012696 PMC2938348

[phy212785-bib-0012] Hepple, R. T. 2014 Mitochondrial involvement and impact in aging skeletal muscle. Front. Aging Neurosci. 6:211. 25309422 10.3389/fnagi.2014.00211 PMC4159998

[phy212785-bib-0013] Herbst, E. A. , S. Paglialunga , C. Gerling , J. Whitfield , K. Mukai , A. Chabowski , et al. 2014 Omega‐3 supplementation alters mitochondrial membrane composition and respiration kinetics in human skeletal muscle. J. Physiol. 592:1341–1352. 24396061 10.1113/jphysiol.2013.267336 PMC3961091

[phy212785-bib-0014] Hinds, S. , W. Bian , R. G. Dennis , and N. Bursac . 2011 The role of extracellular matrix composition in structure and function of bioengineered skeletal muscle. Biomaterials 32:3575–3583. 21324402 10.1016/j.biomaterials.2011.01.062 PMC3057410

[phy212785-bib-0015] Hiona, A. , and C. Leeuwenburgh . 2008 The role of mitochondrial DNA mutations in aging and sarcopenia: implications for the mitochondrial vicious cycle theory of aging. Exp. Gerontol. 43:24–33. 17997255 10.1016/j.exger.2007.10.001 PMC2225597

[phy212785-bib-0016] Huang, F. , H. Wei , H. Luo , S. Jiang , and J. Peng . 2011 EPA inhibits the inhibitor of kappaBalpha (IkappaBalpha)/NF‐kappaB/muscle RING finger 1 pathway in C2C12 myotubes in a PPARgamma‐dependent manner. Br. J. Nutr. 105:348–356. 20955633 10.1017/S0007114510003703

[phy212785-bib-0017] Hyldahl, R. D. , B. Nelson , L. Xin , T. Welling , L. Groscost , M. J. Hubal , et al. 2015 Extracellular matrix remodeling and its contribution to protective adaptation following lengthening contractions in human muscle. FASEB J. 29:2894–2904. 25808538 10.1096/fj.14-266668

[phy212785-bib-0018] Ibebunjo, C. , J. M. Chick , T. Kendall , J. K. Eash , C. Li , Y. Zhang , et al. 2013 Genomic and proteomic profiling reveals reduced mitochondrial function and disruption of the neuromuscular junction driving rat sarcopenia. Mol. Cell. Biol. 33:194–212. 23109432 10.1128/MCB.01036-12 PMC3554128

[phy212785-bib-0019] Johnson, M. L. , A. Z. Lalia , S. Dasari , M. Pallauf , M. Fitch , M. K. Hellerstein , et al. 2015 Eicosapentaenoic acid but not docosahexaenoic acid restores skeletal muscle mitochondrial oxidative capacity in old mice. Aging Cell 14:734–743. 26010060 10.1111/acel.12352 PMC4568961

[phy212785-bib-0020] Kawabata, F. , M. Neya , K. Hamazaki , Y. Watanabe , S. Kobayashi , and T. Tsuji . 2014 Supplementation with eicosapentaenoic acid‐rich fish oil improves exercise economy and reduces perceived exertion during submaximal steady‐state exercise in normal healthy untrained men. Biosci. Biotechnol. Biochem. 78:2081–2088. 25144572 10.1080/09168451.2014.946392

[phy212785-bib-0021] Kim, S. Y. , and D. J. Volsky . 2005 PAGE: parametric analysis of gene set enrichment. BMC Bioinformatics 6:144. 15941488 10.1186/1471-2105-6-144 PMC1183189

[phy212785-bib-0022] Lanza, I. R. , A. Blachnio‐Zabielska , M. L. Johnson , J. M. Schimke , D. R. Jakaitis , N. K. Lebrasseur , et al. 2013 Influence of fish oil on skeletal muscle mitochondrial energetics and lipid metabolites during high‐fat diet. Am. J. Physiol. Endocrinol. Metab. 304:E1391–E1403. 23632634 10.1152/ajpendo.00584.2012 PMC4116354

[phy212785-bib-0023] Lecker, S. H. , R. T. Jagoe , A. Gilbert , M. Gomes , V. Baracos , J. Bailey , et al. 2004 Multiple types of skeletal muscle atrophy involve a common program of changes in gene expression. FASEB J. 18:39–51. 14718385 10.1096/fj.03-0610com

[phy212785-bib-0024] Liu, Y. , F. Chen , J. Odle , X. Lin , H. Zhu , H. Shi , et al. 2013 Fish oil increases muscle protein mass and modulates Akt/FOXO, TLR4, and NOD signaling in weanling piglets after lipopolysaccharide challenge. J. Nutr. 143:1331–1339. 23739309 10.3945/jn.113.176255

[phy212785-bib-0025] Lukashev, M. E. , and Z. Werb . 1998 ECM signalling: orchestrating cell behaviour and misbehaviour. Trends Cell Biol. 8:437–441. 9854310 10.1016/s0962-8924(98)01362-2

[phy212785-bib-0026] Mizunoya, W. , Y. Iwamoto , B. Shirouchi , M. Sato , Y. Komiya , F. R. Razin , et al. 2013 Dietary fat influences the expression of contractile and metabolic genes in rat skeletal muscle. PLoS ONE 8:e80152. 24244634 10.1371/journal.pone.0080152 PMC3823866

[phy212785-bib-0027] Ogasawara, R. , K. Nakazato , K. Sato , M. D. Boppart , and S. Fujita . 2014 Resistance exercise increases active MMP and beta1‐integrin protein expression in skeletal muscle. Physiol. Rep. 2:e12212. 25413329 10.14814/phy2.12212 PMC4255818

[phy212785-bib-0028] Osses, N. , and E. Brandan . 2002 ECM is required for skeletal muscle differentiation independently of muscle regulatory factor expression. Am. J. Physiol. Cell Physiol. 282:C383–C394. 11788350 10.1152/ajpcell.00322.2001

[phy212785-bib-0029] Peoples, G. E. , and P. L. McLennan . 2010 Dietary fish oil reduces skeletal muscle oxygen consumption, provides fatigue resistance and improves contractile recovery in the rat in vivo hindlimb. Br. J. Nutr. 104:1771–1779. 20691135 10.1017/S0007114510002928

[phy212785-bib-0030] Peoples, G. E. , and P. L. McLennan . 2014 Long‐chain n‐3 DHA reduces the extent of skeletal muscle fatigue in the rat in vivo hindlimb model. Br. J. Nutr. 111:996–1003. 24229620 10.1017/S0007114513003449

[phy212785-bib-0031] Peoples, G. E. , P. L. McLennan , P. R. Howe , and H. Groeller . 2008 Fish oil reduces heart rate and oxygen consumption during exercise. J. Cardiovasc. Pharmacol. 52:540–547. 19034030 10.1097/FJC.0b013e3181911913

[phy212785-bib-0032] Philp, L. K. , L. K. Hellborn , A. Janovska , and G. A. Wittert . 2015 Dietary enrichment with fish oil prevents high fat‐induced metabolic dysfunction in skeletal muscle in mice. PLoS ONE 10:e0117494. 25658742 10.1371/journal.pone.0117494 PMC4320112

[phy212785-bib-0033] Rodacki, C. L. , A. L. Rodacki , G. Pereira , K. Naliwaiko , I. Coelho , D. Pequito , et al. 2012 Fish‐oil supplementation enhances the effects of strength training in elderly women. Am. J. Clin. Nutr. 95:428–436. 22218156 10.3945/ajcn.111.021915

[phy212785-bib-0034] Smith, H. J. , S. M. Wyke , and M. J. Tisdale . 2004 Mechanism of the attenuation of proteolysis‐inducing factor stimulated protein degradation in muscle by beta‐hydroxy‐beta‐methylbutyrate. Cancer Res. 64:8731–8735. 15574784 10.1158/0008-5472.CAN-04-1760

[phy212785-bib-0035] Smith, H. J. , J. Khal , and M. J. Tisdale . 2005 Downregulation of ubiquitin‐dependent protein degradation in murine myotubes during hyperthermia by eicosapentaenoic acid. Biochem. Biophys. Res. Commun. 332:83–88. 15896302 10.1016/j.bbrc.2005.04.097

[phy212785-bib-0036] Smith, G. I. , P. Atherton , D. N. Reeds , B. S. Mohammed , D. Rankin , M. J. Rennie , et al. 2011a Dietary omega‐3 fatty acid supplementation increases the rate of muscle protein synthesis in older adults: a randomized controlled trial. Am. J. Clin. Nutr. 93:402–412. 21159787 10.3945/ajcn.110.005611 PMC3021432

[phy212785-bib-0037] Smith, G. I. , P. Atherton , D. N. Reeds , B. S. Mohammed , D. Rankin , M. J. Rennie , et al. 2011b Omega‐3 polyunsaturated fatty acids augment the muscle protein anabolic response to hyperinsulinaemia‐hyperaminoacidaemia in healthy young and middle‐aged men and women. Clin. Sci. 121:267–278. 21501117 10.1042/CS20100597 PMC3499967

[phy212785-bib-0038] Smith, G. I. , J. Yoshino , D. N. Reeds , D. Bradley , R. E. Burrows , H. D. Heisey , et al. 2014 Testosterone and progesterone, but not estradiol, stimulate muscle protein synthesis in postmenopausal women. J. Clin. Endocrinol. Metab. 99:256–265. 24203065 10.1210/jc.2013-2835 PMC3879672

[phy212785-bib-0039] Smith, G. I. , S. Julliand , D. N. Reeds , D. R. Sinacore , S. Klein , and B. Mittendorfer . 2015 Fish oil‐derived n‐3 PUFA therapy increases muscle mass and function in healthy older adults. Am. J. Clin. Nutr. 102:115–122. 25994567 10.3945/ajcn.114.105833 PMC4480667

[phy212785-bib-0040] Supinski, G. S. , J. Vanags , and L. A. Callahan . 2010 Eicosapentaenoic acid preserves diaphragm force generation following endotoxin administration. Crit. Care 14:R35. 20233404 10.1186/cc8913 PMC2887142

[phy212785-bib-0041] Thomas, K. , A. J. Engler , and G. A. Meyer . 2015 Extracellular matrix regulation in the muscle satellite cell niche. Connect. Tissue Res. 56:1–8. 25047058 10.3109/03008207.2014.947369 PMC4464813

[phy212785-bib-0042] Urso, M. L. , A. G. Scrimgeour , Y. W. Chen , P. D. Thompson , and P. M. Clarkson . 2006 Analysis of human skeletal muscle after 48 h immobilization reveals alterations in mRNA and protein for extracellular matrix components. J. Appl. Physiol. 101:1136–1148. 16763108 10.1152/japplphysiol.00180.2006

[phy212785-bib-0043] Vaughan, R. A. , R. Garcia‐Smith , M. Bisoffi , C. A. Conn , and K. A. Trujillo . 2012 Conjugated linoleic acid or omega 3 fatty acids increase mitochondrial biosynthesis and metabolism in skeletal muscle cells. Lipids Health Dis. 11:142. 23107305 10.1186/1476-511X-11-142 PMC3515476

[phy212785-bib-0044] Whitehouse, A. S. , and M. J. Tisdale . 2001 Downregulation of ubiquitin‐dependent proteolysis by eicosapentaenoic acid in acute starvation. Biochem. Biophys. Res. Commun. 285:598–602. 11453634 10.1006/bbrc.2001.5209

[phy212785-bib-0045] Whitehouse, A. S. , H. J. Smith , J. L. Drake , and M. J. Tisdale . 2001 Mechanism of attenuation of skeletal muscle protein catabolism in cancer cachexia by eicosapentaenoic acid. Cancer Res. 61:3604–3609. 11325828

[phy212785-bib-0046] Whitehouse, A. S. , J. Khal , and M. J. Tisdale . 2003 Induction of protein catabolism in myotubes by 15(S)‐hydroxyeicosatetraenoic acid through increased expression of the ubiquitin‐proteasome pathway. Br. J. Cancer 89:737–745. 12915888 10.1038/sj.bjc.6601184 PMC2376908

[phy212785-bib-0047] Yoshino, J. , K. F. Mills , M. J. Yoon , and S. Imai . 2011 Nicotinamide mononucleotide, a key NAD(+) intermediate, treats the pathophysiology of diet‐ and age‐induced diabetes in mice. Cell Metab. 14:528–536. 21982712 10.1016/j.cmet.2011.08.014 PMC3204926

[phy212785-bib-0048] Yoshino, J. , P. Almeda‐Valdes , B. W. Patterson , A. L. Okunade , S. Imai , B. Mittendorfer , et al. 2014 Diurnal variation in insulin sensitivity of glucose metabolism is associated with diurnal variations in whole‐body and cellular fatty acid metabolism in metabolically normal women. J. Clin. Endocrinol. Metab. 99:E1666–E1670. 24878055 10.1210/jc.2014-1579 PMC4154096

[phy212785-bib-0049] Zhang, Q. , S. K. Joshi , D. H. Lovett , B. Zhang , S. Bodine , H. T. Kim , et al. 2015 Matrix metalloproteinase‐2 plays a critical role in overload induced skeletal muscle hypertrophy. Muscles Ligaments Tendons J. 4:446–454. 25767782 PMC4327354

