# Supplementary material for: Effect of dietary n‐3 PUFA supplementation on the muscle transcriptome in older adults
Source: Physiol Rep. 2016 Jun 1;4(11):e12785. doi: 10.14814/phy2.12785 (PMC4908485; doi:10.14814/phy2.12785)
Supplement: Supplementary file 1 — Table S1. Additional gene set pathways in skeletal muscle that were significantly changed by n‐3 PUFA therapy. [file PHY2-4-e12785-s001.docx]

**Online supporting information**

**Additional gene set pathways in skeletal muscle that were significantly changed by n-3 PUFA therapy.**

| Gene set name | Z score | P-value |
| --- | --- | --- |
| BIOCARTA_AHSP_PATHWAY | 8.14 | 4.44E-16 |
| REACTOME_G_ALPHA_I_SIGNALLING_EVENTS | 5.47 | 4.39E-08 |
| BIOCARTA_TCRA_PATHWAY | 4.65 | 3.38E-06 |
| REACTOME_CLASS_A1_RHODOPSIN_LIKE_RECEPTORS | 4.33 | 1.52E-05 |
| REACTOME_CHEMOKINE_RECEPTORS_BIND_CHEMOKINES | 4.12 | 3.81E-05 |
| REACTOME_PD1_SIGNALING | 4.09 | 4.30E-05 |
| REACTOME_SIGNAL_REGULATORY_PROTEIN_SIRP_FAMILY_INTERACTIONS | 4.00 | 6.46E-05 |
| KEGG_PRIMARY_IMMUNODEFICIENCY | 3.69 | 2.28E-04 |
| BIOCARTA_CCR5_PATHWAY | 3.68 | 2.35E-04 |
| BIOCARTA_THELPER_PATHWAY | 3.64 | 2.76E-04 |
| REACTOME_PEPTIDE_LIGAND_BINDING_RECEPTORS | 3.63 | 2.88E-04 |
| SIG_PIP3_SIGNALING_IN_B_LYMPHOCYTES | 3.57 | 3.58E-04 |
| REACTOME_IMMUNOREGULATORY_INTERACTIONS_BETWEEN_A_LYMPHOID_AND_A_NON_LYMPHOID_CELL | 3.55 | 3.82E-04 |
| REACTOME_PHOSPHORYLATION_OF_CD3_AND_TCR_ZETA_CHAINS | 3.43 | 6.01E-04 |
| REACTOME_TRANSLOCATION_OF_ZAP_70_TO_IMMUNOLOGICAL_SYNAPSE | 3.34 | 8.27E-04 |
| KEGG_CYTOKINE_CYTOKINE_RECEPTOR_INTERACTION | 3.06 | 2.22E-03 |
| REACTOME_GENERATION_OF_SECOND_MESSENGER_MOLECULES | 3.06 | 2.22E-03 |
| REACTOME_HEMOSTASIS | 3.05 | 2.25E-03 |
| KEGG_GRAFT_VERSUS_HOST_DISEASE | 2.93 | 3.36E-03 |
| BIOCARTA_TCAPOPTOSIS_PATHWAY | 2.91 | 3.59E-03 |
| KEGG_INTESTINAL_IMMUNE_NETWORK_FOR_IGA_PRODUCTION | 2.89 | 3.89E-03 |
| BIOCARTA_VITCB_PATHWAY | 2.86 | 4.29E-03 |
| KEGG_NEUROACTIVE_LIGAND_RECEPTOR_INTERACTION | 2.80 | 5.07E-03 |
| BIOCARTA_TCYTOTOXIC_PATHWAY | 2.80 | 5.07E-03 |
| REACTOME_POTASSIUM_CHANNELS | 2.80 | 5.15E-03 |
| KEGG_CHEMOKINE_SIGNALING_PATHWAY | 2.71 | 6.65E-03 |
| REACTOME_AMINO_ACID_SYNTHESIS_AND_INTERCONVERSION_TRANSAMINATION | 2.71 | 6.66E-03 |
| ST_GRANULE_CELL_SURVIVAL_PATHWAY | 2.67 | 7.53E-03 |
| KEGG_HEMATOPOIETIC_CELL_LINEAGE | 2.66 | 7.93E-03 |
| BIOCARTA_CTL_PATHWAY | 2.65 | 7.95E-03 |
| BIOCARTA_NO2IL12_PATHWAY | 2.60 | 9.37E-03 |
| REACTOME_EICOSANOID_LIGAND_BINDING_RECEPTORS | 2.48 | 1.30E-02 |
| KEGG_PARKINSONS_DISEASE | 2.47 | 1.35E-02 |
| KEGG_ALZHEIMERS_DISEASE | 2.47 | 1.35E-02 |
| REACTOME_GLUCONEOGENESIS | 2.47 | 1.36E-02 |
| BIOCARTA_GRANULOCYTES_PATHWAY | 2.45 | 1.41E-02 |
| KEGG_TYPE_I_DIABETES_MELLITUS | 2.45 | 1.43E-02 |
| SA_MMP_CYTOKINE_CONNECTION | 2.44 | 1.46E-02 |
| REACTOME_DEFENSINS | 2.36 | 1.84E-02 |
| REACTOME_FGFR4_LIGAND_BINDING_AND_ACTIVATION | 2.34 | 1.93E-02 |
| KEGG_HUNTINGTONS_DISEASE | 2.34 | 1.93E-02 |
| REACTOME_GPVI_MEDIATED_ACTIVATION_CASCADE | 2.33 | 1.99E-02 |
| ST_TUMOR_NECROSIS_FACTOR_PATHWAY | 2.31 | 2.07E-02 |
| KEGG_FOLATE_BIOSYNTHESIS | 2.31 | 2.08E-02 |
| REACTOME_OLFACTORY_SIGNALING_PATHWAY | 2.30 | 2.15E-02 |
| REACTOME_FACTORS_INVOLVED_IN_MEGAKARYOCYTE_DEVELOPMENT_AND_PLATELET_PRODUCTION | 2.29 | 2.20E-02 |
| KEGG_ALLOGRAFT_REJECTION | 2.25 | 2.45E-02 |
| REACTOME_COSTIMULATION_BY_THE_CD28_FAMILY | 2.23 | 2.56E-02 |
| KEGG_LEUKOCYTE_TRANSENDOTHELIAL_MIGRATION | 2.20 | 2.79E-02 |
| BIOCARTA_CHEMICAL_PATHWAY | 2.19 | 2.85E-02 |
| KEGG_OLFACTORY_TRANSDUCTION | 2.17 | 3.00E-02 |
| BIOCARTA_D4GDI_PATHWAY | 2.14 | 3.20E-02 |
| REACTOME_BINDING_AND_ENTRY_OF_HIV_VIRION | 2.12 | 3.44E-02 |
| REACTOME_ACTIVATION_OF_BH3_ONLY_PROTEINS | 2.11 | 3.48E-02 |
| REACTOME_PASSIVE_TRANSPORT_BY_AQUAPORINS | 2.09 | 3.68E-02 |
| REACTOME_GLUCAGON_TYPE_LIGAND_RECEPTORS | 2.07 | 3.83E-02 |
| ST_B_CELL_ANTIGEN_RECEPTOR | 2.04 | 4.17E-02 |
| KEGG_NITROGEN_METABOLISM | 2.02 | 4.37E-02 |
| REACTOME_FGFR2C_LIGAND_BINDING_AND_ACTIVATION | 1.98 | 4.72E-02 |
| REACTOME_VOLTAGE_GATED_POTASSIUM_CHANNELS | 1.97 | 4.90E-02 |
| REACTOME_CHONDROITIN_SULFATE_BIOSYNTHESIS | 1.97 | 4.91E-02 |
| ST_T_CELL_SIGNAL_TRANSDUCTION | 1.97 | 4.91E-02 |
| REACTOME_SYNTHESIS_OF_PIPS_AT_THE_PLASMA_MEMBRANE | -1.97 | 4.90E-02 |
| BIOCARTA_AGR_PATHWAY | -1.97 | 4.89E-02 |
| REACTOME_S_PHASE | -1.97 | 4.86E-02 |
| KEGG_LYSINE_DEGRADATION | -1.98 | 4.81E-02 |
| REACTOME_ACTIVATED_AMPK_STIMULATES_FATTY_ACID_OXIDATION_IN_MUSCLE | -1.98 | 4.79E-02 |
| REACTOME_THE_ACTIVATION_OF_ARYLSULFATASES | -1.98 | 4.75E-02 |
| REACTOME_PTM_GAMMA_CARBOXYLATION_HYPUSINE_FORMATION_AND_ARYLSULFATASE_ACTIVATION | -1.98 | 4.72E-02 |
| REACTOME_TRNA_AMINOACYLATION | -1.99 | 4.62E-02 |
| REACTOME_SIGNALING_BY_ERBB4 | -2.00 | 4.51E-02 |
| REACTOME_METABOLISM_OF_LIPIDS_AND_LIPOPROTEINS | -2.02 | 4.38E-02 |
| REACTOME_POST_TRANSLATIONAL_MODIFICATION_SYNTHESIS_OF_GPI_ANCHORED_PROTEINS | -2.02 | 4.32E-02 |
| REACTOME_TIE2_SIGNALING | -2.03 | 4.22E-02 |
| KEGG_GLYCOSAMINOGLYCAN_DEGRADATION | -2.03 | 4.21E-02 |
| REACTOME_RNA_POL_III_TRANSCRIPTION | -2.05 | 4.04E-02 |
| KEGG_BIOSYNTHESIS_OF_UNSATURATED_FATTY_ACIDS | -2.06 | 3.93E-02 |
| KEGG_GLUTATHIONE_METABOLISM | -2.07 | 3.87E-02 |
| BIOCARTA_DEATH_PATHWAY | -2.08 | 3.79E-02 |
| KEGG_ONE_CARBON_POOL_BY_FOLATE | -2.09 | 3.70E-02 |
| REACTOME_NUCLEOTIDE_EXCISION_REPAIR | -2.09 | 3.62E-02 |
| REACTOME_TRANSCRIPTION | -2.10 | 3.54E-02 |
| REACTOME_CDT1_ASSOCIATION_WITH_THE_CDC6_ORC_ORIGIN_COMPLEX | -2.11 | 3.52E-02 |
| KEGG_ERBB_SIGNALING_PATHWAY | -2.11 | 3.51E-02 |
| BIOCARTA_RARRXR_PATHWAY | -2.11 | 3.49E-02 |
| BIOCARTA_ARF_PATHWAY | -2.11 | 3.49E-02 |
| BIOCARTA_LEPTIN_PATHWAY | -2.12 | 3.40E-02 |
| REACTOME_3_UTR_MEDIATED_TRANSLATIONAL_REGULATION | -2.13 | 3.32E-02 |
| REACTOME_BIOLOGICAL_OXIDATIONS | -2.13 | 3.31E-02 |
| REACTOME_MITOTIC_G1_G1_S_PHASES | -2.14 | 3.26E-02 |
| REACTOME_NONSENSE_MEDIATED_DECAY_ENHANCED_BY_THE_EXON_JUNCTION_COMPLEX | -2.14 | 3.25E-02 |
| REACTOME_CASPASE_MEDIATED_CLEAVAGE_OF_CYTOSKELETAL_PROTEINS | -2.14 | 3.21E-02 |
| REACTOME_PPARA_ACTIVATES_GENE_EXPRESSION | -2.15 | 3.17E-02 |
| REACTOME_SIGNALING_BY_ERBB2 | -2.15 | 3.14E-02 |
| REACTOME_DOWNSTREAM_SIGNAL_TRANSDUCTION | -2.15 | 3.12E-02 |
| REACTOME_SIGNALING_BY_WNT | -2.16 | 3.06E-02 |
| REACTOME_SIGNALING_BY_FGFR_IN_DISEASE | -2.18 | 2.89E-02 |
| REACTOME_HORMONE_SENSITIVE_LIPASE_HSL_MEDIATED_TRIACYLGLYCEROL_HYDROLYSIS | -2.19 | 2.87E-02 |
| REACTOME_CYCLIN_E_ASSOCIATED_EVENTS_DURING_G1_S_TRANSITION_ | -2.24 | 2.53E-02 |
| REACTOME_FORMATION_OF_TRANSCRIPTION_COUPLED_NER_TC_NER_REPAIR_COMPLEX | -2.25 | 2.46E-02 |
| KEGG_TASTE_TRANSDUCTION | -2.25 | 2.44E-02 |
| REACTOME_MRNA_SPLICING_MINOR_PATHWAY | -2.25 | 2.42E-02 |
| REACTOME_MRNA_3_END_PROCESSING | -2.26 | 2.39E-02 |
| BIOCARTA_CREB_PATHWAY | -2.26 | 2.39E-02 |
| REACTOME_SIGNALING_BY_CONSTITUTIVELY_ACTIVE_EGFR | -2.26 | 2.36E-02 |
| REACTOME_KERATAN_SULFATE_DEGRADATION | -2.27 | 2.32E-02 |
| KEGG_CHRONIC_MYELOID_LEUKEMIA | -2.29 | 2.23E-02 |
| REACTOME_APOPTOTIC_EXECUTION_PHASE | -2.29 | 2.21E-02 |
| REACTOME_ASSOCIATION_OF_TRIC_CCT_WITH_TARGET_PROTEINS_DURING_BIOSYNTHESIS | -2.30 | 2.16E-02 |
| REACTOME_FORMATION_OF_INCISION_COMPLEX_IN_GG_NER | -2.32 | 2.04E-02 |
| REACTOME_REGULATION_OF_RHEB_GTPASE_ACTIVITY_BY_AMPK | -2.33 | 2.00E-02 |
| REACTOME_SIGNALING_BY_EGFR_IN_CANCER | -2.35 | 1.87E-02 |
| BIOCARTA_GATA3_PATHWAY | -2.36 | 1.81E-02 |
| KEGG_METABOLISM_OF_XENOBIOTICS_BY_CYTOCHROME_P450 | -2.36 | 1.81E-02 |
| REACTOME_REGULATION_OF_AMPK_ACTIVITY_VIA_LKB1 | -2.37 | 1.77E-02 |
| REACTOME_TRANSPORT_OF_MATURE_TRANSCRIPT_TO_CYTOPLASM | -2.38 | 1.73E-02 |
| KEGG_AMINOACYL_TRNA_BIOSYNTHESIS | -2.40 | 1.64E-02 |
| REACTOME_G1_S_TRANSITION | -2.41 | 1.61E-02 |
| REACTOME_PKA_MEDIATED_PHOSPHORYLATION_OF_CREB | -2.42 | 1.56E-02 |
| REACTOME_CYTOSOLIC_TRNA_AMINOACYLATION | -2.43 | 1.51E-02 |
| REACTOME_IRAK1_RECRUITS_IKK_COMPLEX | -2.47 | 1.37E-02 |
| BIOCARTA_PPARA_PATHWAY | -2.48 | 1.31E-02 |
| REACTOME_RNA_POL_I_TRANSCRIPTION_INITIATION | -2.53 | 1.15E-02 |
| REACTOME_DEADENYLATION_OF_MRNA | -2.54 | 1.10E-02 |
| REACTOME_CIRCADIAN_REPRESSION_OF_EXPRESSION_BY_REV_ERBA | -2.55 | 1.09E-02 |
| KEGG_RNA_DEGRADATION | -2.55 | 1.06E-02 |
| REACTOME_RNA_POL_I_TRANSCRIPTION_TERMINATION | -2.55 | 1.06E-02 |
| REACTOME_ANTIVIRAL_MECHANISM_BY_IFN_STIMULATED_GENES | -2.56 | 1.05E-02 |
| REACTOME_NFKB_ACTIVATION_THROUGH_FADD_RIP1_PATHWAY_MEDIATED_BY_CASPASE_8_AND10 | -2.56 | 1.05E-02 |
| REACTOME_GENERIC_TRANSCRIPTION_PATHWAY | -2.57 | 1.01E-02 |
| KEGG_PHOSPHATIDYLINOSITOL_SIGNALING_SYSTEM | -2.58 | 9.84E-03 |
| REACTOME_PKB_MEDIATED_EVENTS | -2.59 | 9.56E-03 |
| REACTOME_MICRORNA_MIRNA_BIOGENESIS | -2.60 | 9.36E-03 |
| REACTOME_SIGNALING_BY_FGFR1_FUSION_MUTANTS | -2.60 | 9.33E-03 |

Positive Z-scores indicate an upregulation (increased gene expression) and negative values a downregulation (decreased gene expression) of pathways.
